# Endoscopic closure of cough-induced esophageal rupture during endoscopic submucosal dissection using the reopenable clip-over-the-line method

**DOI:** 10.1055/a-2945-3401

**Published:** 2026-09-03

**Authors:** Tomohiro Shimada, Taku Yamagata, Yoshihide Kanno, Takeshi Shimizu, Kai Korekawa, Hiroki Sato, Kei Ito

**Affiliations:** 1 Department of GastroenterologySendai City Medical CenterSendaiJapan

**Fig. 1:**
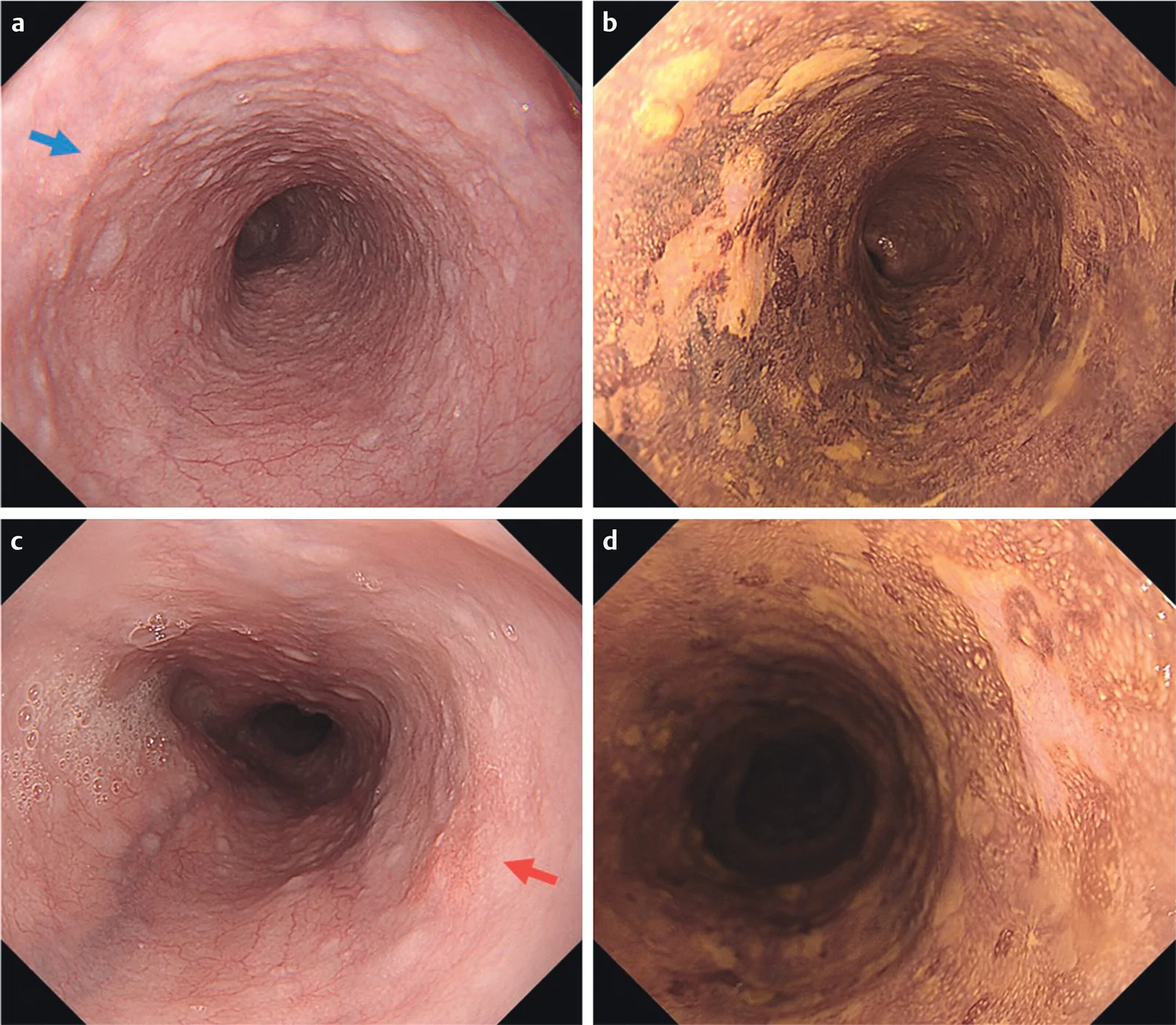
Two synchronous early esophageal cancers. ( **a** and **b** ) A 0-IIc, 25-mm lesion in the left wall of the middle thoracic esophagus (blue arrow). ( **c** and **d** ) A 0-IIc, 10-mm lesion in the right wall of the upper thoracic esophagus (red arrow).

**Fig. 2:**
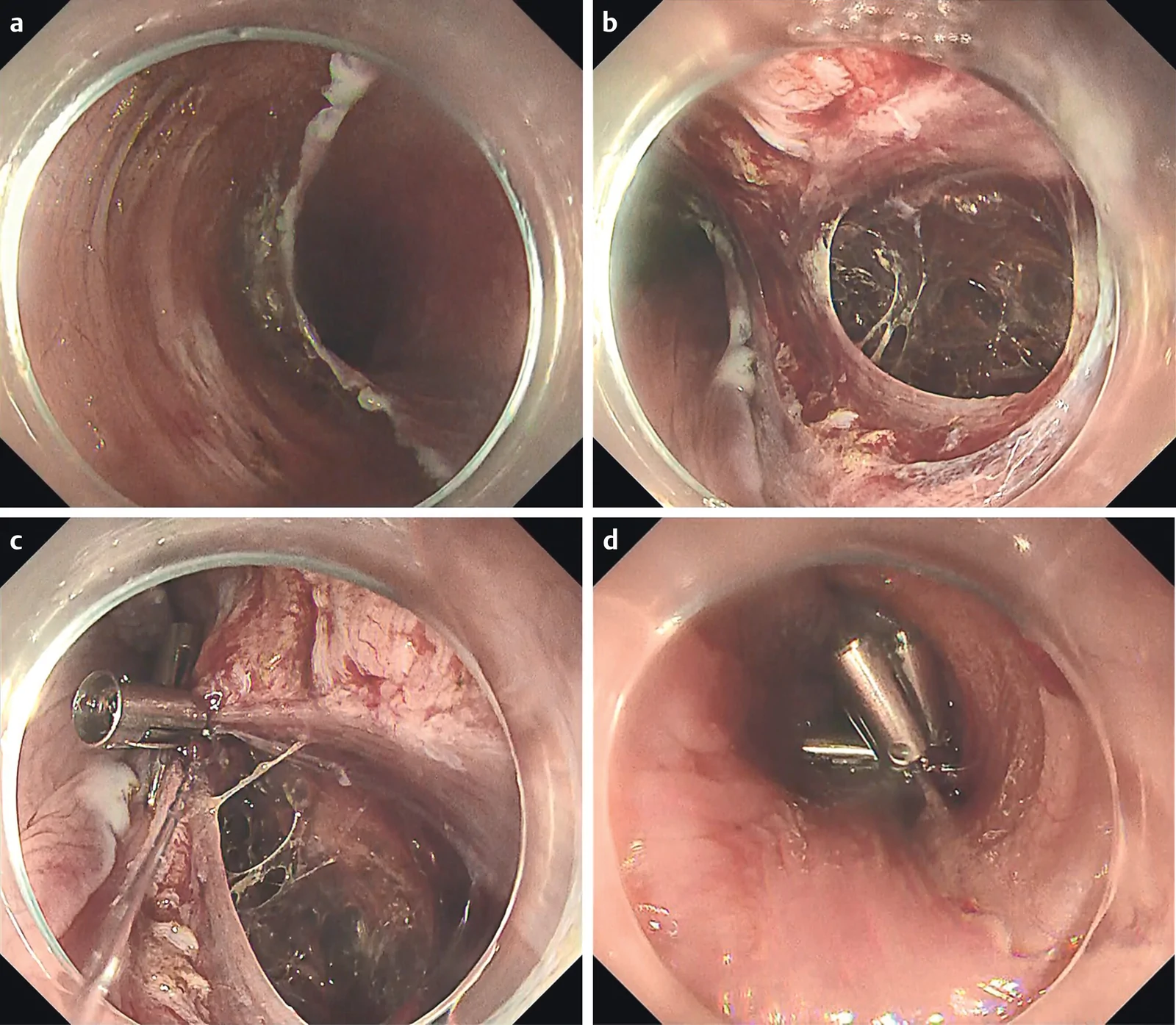
Endoscopic closure of a large perforation in the post-ESD mucosal defect in the middle thoracic esophagus using the reopenable clip-over-the-line method (ROLM). ( **a** ) Post-ESD mucosal defect without evidence of perforation. ( **b** ) Large perforation at the post-ESD mucosal defect. ( **c** and **d** ) Endoscopic closure using ROLM. ESD, endoscopic submucosal dissection.

**Fig. 3:**
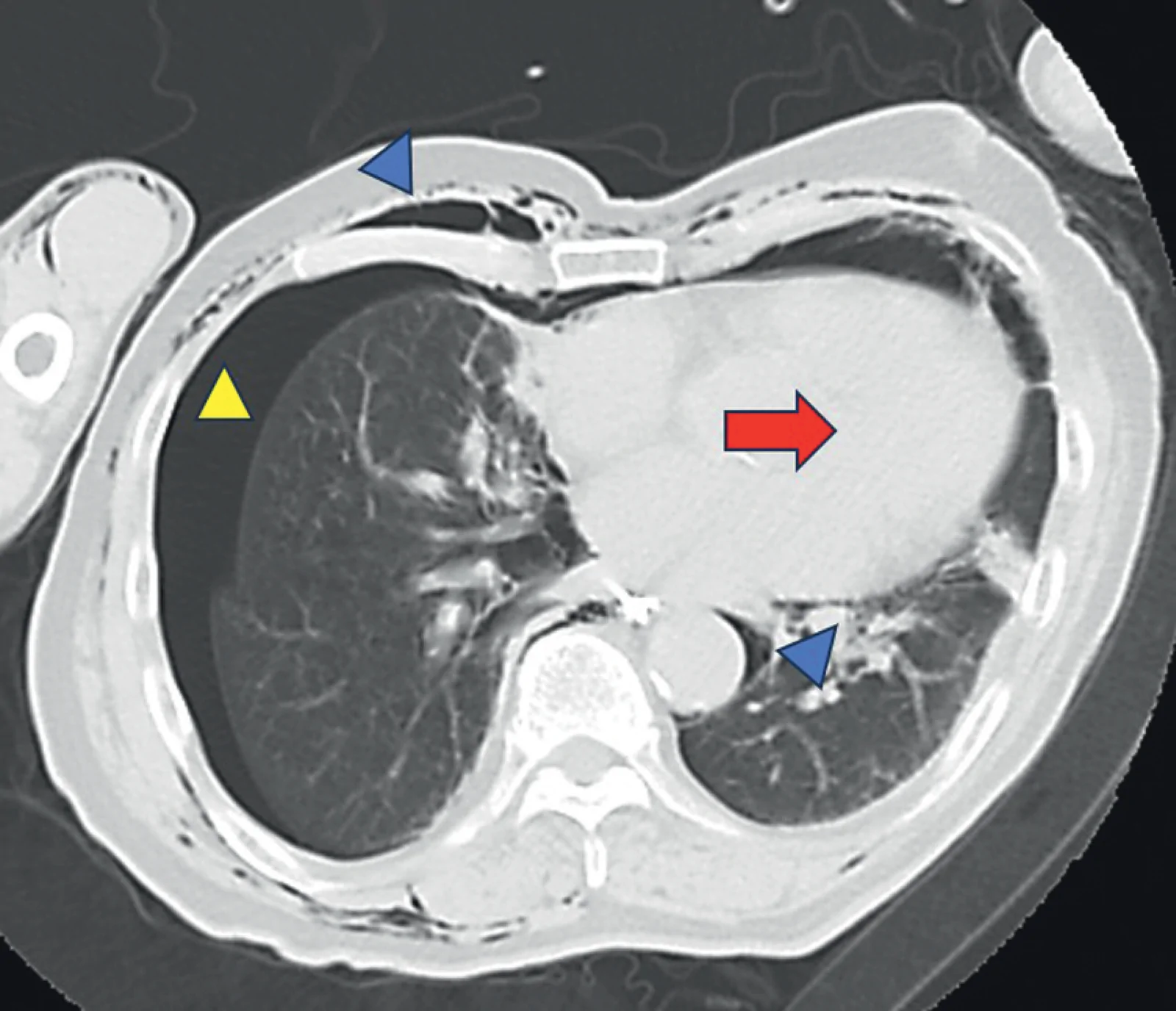
CT performed immediately after endoscopic submucosal dissection. By using CT, extensive subcutaneous and mediastinal emphysema (blue arrowheads) and a pneumothorax (yellow arrowhead) with a mediastinal shift (red arrow) were revealed. CT, computed tomography.

**Fig. 4:**
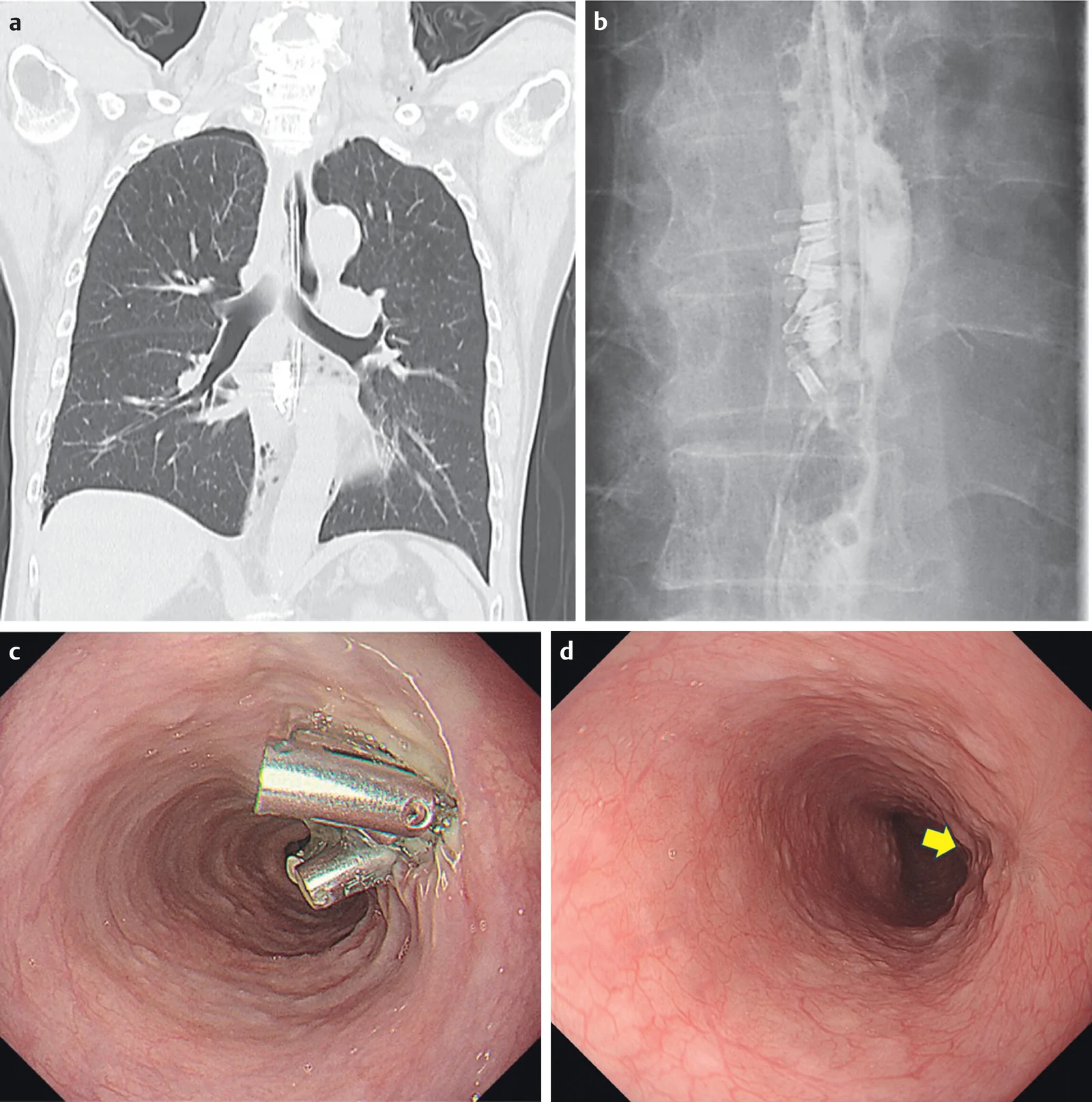
Follow-up examinations. ( **a** and **b** ) Computed tomography and esophagography on postprocedural day 7. The images revealed that the emphysema, pneumothorax, and mediastinal shift were resolved with no leakage of the contrast medium from the closure site. ( **c** and **d** ) Endoscopy performed on day 13 and 2 months later revealed a progressively healing ulcer and subsequent complete healing with scarring (yellow arrow), respectively.

A 74-year-old man with two synchronous early cancers in the upper and middle esophagus underwent same-session endoscopic submucosal dissection (ESD; Fig. 1 ). The procedure was performed under deep sedation using continuous propofol infusion and pentazocine boluses. The middle esophageal lesion was resected first without evidence of perforation, and subsequent resection of the upper esophageal lesion was similarly uneventful. Immediately after the completion of the second ESD, re-inspection of the initially resected ulcer revealed a large perforation in the middle thoracic esophagus ( Fig. 2 ). Anesthesia records showed a sudden surge in blood pressure requiring additional administration of sedatives and analgesics, and a review of the procedural video confirmed patient movement and severe coughing during upper esophageal ESD. Although the moment of perforation was not directly visualized, the findings indicated Boerhaave-like barotrauma caused by cough-reflex-induced elevation of intraesophageal pressure. 1​ Because of the large size of the perforation, the defect was closed using the reopenable clip-over-line method (ROLM) with sure-clips (Microtech, China) and a 3-0 nylon line, 2​ as previously reported 3​ (Supplementary **Video S1** , available in the online version only). Postprocedural computed tomography (CT) revealed extensive subcutaneous and mediastinal emphysema and a pneumothorax with a mediastinal shift ( Fig. 3 ). However, the patient remained hemodynamically stable and was managed conservatively with antibiotics and transnasal esophageal tube decompression. On postprocedural day 7, CT revealed the resolution of emphysema, pneumothorax, and mediastinal shift. Esophagography showed no evidence of contrast leakage, allowing the resumption of oral intake. Follow-up endoscopy on day 13 confirmed the closure of the perforation with a stable healing ulcer, and the patient was discharged. At the 2-month follow-up, the perforation site had completely healed with scarring ( Fig. 4 ).

Esophageal rupture from coughing during ESD is rare but can occur in any case, becoming potentially life-threatening. In hemodynamically stable patients, endoscopic closure using ROLM may serve as an effective, minimally invasive alternative to emergency surgery ( Video 1 ).

**Video 1** Endoscopic closure of a large perforation at the post-endoscopic submucosal dissection (ESD) mucosal defect in the middle thoracic esophagus using a reopenable-clip over-the-line method (ROLM). .

